# Pre-Study protocol MagPEP: a multicentre randomized controlled trial of magnesium sulphate in the prevention of post-ERCP pancreatitis

**DOI:** 10.1186/1471-230X-13-11

**Published:** 2013-01-15

**Authors:** Gabriele Fluhr, Julia Mayerle, Eckhard Weber, Ali Aghdassi, Peter Simon, Thomas Gress, Thomas Seufferlein, Joachim Mössner, Andreas Stallmach, Thomas Rösch, Martina Müller, Britta Siegmund, Petra Büchner-Steudel, Ina Zuber-Jerger, Marcus Kantowski, Albrecht Hoffmeister, Jonas Rosendahl, Thomas Linhart, Jochen Maul, László Czakó, Péter Hegyi, Matthias Kraft, Georg Engel, Thomas Kohlmann, Anne Glitsch, Tilman Pickartz, Christoph Budde, Claudia Nitsche, Kirsten Storck, Markus M Lerch

**Affiliations:** 1 Central Endoscopy and Department of Medicine A, University Medicine Greifswald, Ferdinand-Sauerbruch-Straße, Greifswald, 17475, Germany; 2 Division of Gastroenterology, Endocrinology and Metabolism, Department of Medicine, Philipps-Universität Marburg, Baldingerstraße, Marburg, 35043, Germany; 3 Department of Medicine I, University Hospital Ulm, Albert-Einstein-Allee 23, Ulm, 89070, Germany; 4 Division of Gastroenterology and Rheumatology, Department of Medicine, Neurology and Dermatology, University Hospital Leipzig AöR, Liebigstr. 20, Leipzig, 04103, Germany; 5 Division of Gastroenterology, Hepatology and Infectious Diseases, Department of Medicine II, University Hospital Jena, Erlanger Allee 101, Jena, 07743, Germany; 6 Department of Interdisciplinary Endoscopy, University Hospital Eppendorf, Martinistr. 52, Hamburg, 20246, Germany; 7 Department of Internal Medicine I, University Hospital Regensburg, Franz-Joseph-Strauß-Allee 11, Regensburg, 93042, Germany; 8 Department for Gastroenterology, Infectious Diseases and Rheumatology, Charité – University Medicine, Campus Benjamin Franklin, Hindenburgdamm 30, Berlin, 12203, Germany; 9 Department of Medicine I, University Hospital Halle, Ernst-Grube-Str. 40, Halle, 06120, Germany; 10 First Department of Medicine, University of Szeged, 6701 Szeged, P.O.Box: 427, Szeged, Hungary; 11 University Pharmacy, University Medicine Greifswald, Friedrich-Ludwig-Jahn-Straße 20, Greifswald, 17475, Germany; 12 Department of Methods in Community Medicinem Institute for Community Medicine, University Medicine Greifswald, Walther-Rathenau-Str, Greifswald, 4817475, Germany; 13 Central Endoscopy and Department of Surgery, University Medicine Greifswald, Ferdinand-Sauerbruch-Straße, Greifswald, 17475, Germany

## Abstract

**Background:**

Acute pancreatitis is the most common complication of diagnostic and therapeutic endoscopic retrograde cholangiopancreatography (ERCP). In spite of continuing research, no pharmacologic agent capable of effectively reducing the incidence of ERCP-induced pancreatitis has found its way into clinical practise. A number of experimental studies suggest that intrapancreatic calcium concentrations play an important role in the initiation of intracellular protease activation, an initiating step in the course of acute pancreatitis. Magnesium can act as a calcium-antagonist and counteracts effects in calcium signalling. It can thereby attenuate the intracellular activation of proteolytic digestive enzymes in the pancreas and reduces the severity of experimental pancreatitis when administered either intravenously or as a food supplement.

**Methods:**

We designed a randomized, double-blind, placebo-controlled phase III study to test whether the administration of intravenous magnesium sulphate before and after ERCP reduces the incidence and the severity of post-ERCP pancreatitis. A total of 502 adult patients with a medical indication for ERCP are to be randomized to receive either 4930 mg magnesium sulphate (= 20 mmol magnesium) or placebo 60 min before and 6 hours after ERCP. The incidence of clinical post-ERCP pancreatitis, hyperlipasemia, pain levels, use of analgetics and length of hospital stay will be evaluated.

**Conclusions:**

If magnesium sulphate is found to be effective in preventing post-ERCP pancreatitis, this inexpensive agent with limited adverse effects could be used as a routine pharmacological prophylaxis.

**Trial registration:**

Current Controlled Trials ISRCTN46556454

## Background

Endoscopic retrograde cholangiopancreatography (ERCP) is a common procedure employed to treat disorders of the biliary tract or the pancreas. In Germany around 124.000 ERCP’s (OPS 1–64, diagnostic ERC, ERP or ERCP) are performed annually (German federal statistics bureau for 2009). The most common complication of ERCP is procedure-related acute pancreatitis, which occurs in 2 to 9% of patients in unselected prospective studies [1]. When an average rate of 5% is assumed, 6200 pancreatitis cases in Germany would be triggered by prior ERCP and thus 12% of the total 53.338 in-hospital pancreatitis cases in the country (German federal statistics bureau for 2009). The severity of post-ERCP pancreatitis can range from mild disease with full recovery to critical illness with pancreatic necrosis, multiple organ failure, prolonged hospitalization and even death. Of all cases of post-ERCP pancreatitis approximately 10% are severe and up to 1% take a fatal course [2]. The national in-hospital mortality for acute pancreatitis is 2.9% in Germany and ERCP-induced pancreatitis would thus account for at least 180 deaths.

Efforts to reduce the incidence and severity of post-ERCP pancreatitis have been made for more than thirty years. So far, the search for a pharmacologic agent that would be effective and broadly introduced into clinical routine has been unsuccessful. Some of the pharmacologic agents that have been tested for the prophylactic administration before and during ERCP are gabexate mesilate [3-5], octreotide [6], somatostatin [7,8], allopurinol [9,10], corticosteroids [11], NSAIDs [12], heparin [13] and interleukin-10 [14]. The most promising study suggests the routine use of diclofenac suppositories but has demonstrated its effectiveness only in very high risk patients [15].

One critical event for the development of acute pancreatitis is premature intracellular zymogen activation leading to pancreatic autodigestion [16]. Extra- as well as intracellular calcium concentrations have been shown to play an important role in the initiation of intracellular pancreatic protease activation and the onset of pancreatitis [17,18]. Moreover, pathological sustained calcium signals have been shown to strongly reduce bicarbonate secretion from pancreatic duct cells [19,20], which may be relevant for the initiation of post-ERCP pancreatitis. Magnesium, a critical cofactor for multiple enzymatic reactions, acts as a natural calcium-antagonist in the exocrine pancreas and can counteract the effect of pathological calcium signals on premature intracellular protease activation and cell necrosis [21]. In animal models of acute pancreatitis Mg^2+^ administration not only reduced the intrapancreatic activation of digestive enzymes but also ameliorated the local and systemic damage associated with the disease [22].

Based on these *in vitro* and animal experiments we designed a multicentre, randomized placebo-controlled phase III trial to study the efficacy of magnesium-sulphate in preventing the onset and reducing the severity of acute post-ERCP pancreatitis. The “Magnesium sulphate in the prevention of post-ERCP pancreatitis study” (MagPEP) aims to provide robust data on the effectiveness of peri-interventional intravenous magnesium sulphate administration in reducing the incidence and severity of post-ERCP pancreatitis.

## Methods

### Trial organisation and coordination

MagPEP is designed and coordinated by the Department of Medicine A at University Medicine Greifswald, which is responsible for overall trial management, regulatory affairs, statistical planning and analysis, trial registration and reporting as well as quality assurance. Monitoring is carried out by the Clinical Trial Coordination Centre at Greifswald University Medicine. MagPEP will be conducted as a multicentre trial in Germany and Hungary, including (as of November 2012) ten academic medical centres. The trial is sponsored by the University Hospital Greifswald, represented by the CEO, and the Deutsche Forschungsgemeinschaft (DFG), neither of which is involved in the database management or has access to the randomisation code.

### Investigators

Patients will be recruited by the respective centres who will commit their participation in a contractual agreement with the sponsor. All investigators will be experienced gastroenterologists and endoscopists. Centres participating in the trial will have a volume load of > 400 ERCP per annum.

### Data safety and monitoring board

An independent Data Safety and Monitoring Board (DSMB) consisting of three independent experts will evaluate the clinical research data on an ongoing basis to assure patient safety and study integrity. The board will monitor the trial data, particularly the safety data, and give their advice based on the periodical reviews. Responsibilities are recorded in a DSMB chart.

### Medication supply

Study medication and placebo will be prepared and provided by the university pharmacy in Greifswald which is ICH-GCP approved and audited by the state regulatory authorities.

### On-site monitoring

On-site monitoring of the centres will be performed according to good clinical practice (ICH-GCP) guidelines. In person visits carried out according to the SOPs of the Clinical Trial Coordination Centre at Greifswald University Medicine, clinical monitors will review all entries into CRFs on the basis of source documents (minimum of 30% source data verification) and perform the data management.

### Ethical considerations

The final protocol has been approved by the ethics committee at the Medical Faculty of the Ernst-Moritz-Arndt-University Greifswald on August 27th 2010 (Registration number: FFV 04/10) and adheres to the declaration of Helsinki, the Medical Association’s professional code of conduct, the principles of ICH-GCP guidelines and the Federal Data Protection Act. The trial will also be performed in keeping with local legal and regulatory requirements. The medical secrecy and the Federal Data Protection Act will be followed. Consent to the master ethics committee approval has been obtained from the local ethics committees of all participating centres.

Before enrolment, each patient will be given a detailed briefing on the nature, scope and possible consequences of the trial by a physician and then give written informed consent. No measures required specifically for the clinical trial will be taken without valid consent having been obtained from the patient.

### Study objectives

The primary objective of this study is to determine whether intravenous magnesium sulphate administrated before and after the intervention can reduce the incidence and severity of post-ERCP pancreatitis by 50% i.e. (from 7.4%, the incidence in Greifswald, Germany, in 2004–2005, to 3.7%). Secondary objectives include the evaluation of (1) the severity of post-ERCP pancreatitis, the (2) administration of analgesics, the (3) duration of the hospital stay, the (4) lipase levels measured 6 and 24 hours after ERCP and the (5) 30-days mortality as determined in a telephone interview 30 days after ERCP.

### Definitions

Acute post-ERCP pancreatitis is defined as the presence of typical abdominal pain at 24 hours post ERCP in combination with an elevation in serum lipase level to at least three times the normal upper limit. Severity of pancreatitis is classified according to the 1991 Consensus Guidelines published by Cotton et al. [23]: mild pancreatitis leading to a prolongation of hospitalization by up to three days, moderate pancreatitis requiring four to ten days of hospitalization and severe pancreatitis involving hospitalization for more than ten days and evidence of necrotizing pancreatitis and/or infected necrosis or pseudocyst (fluid collection) development.

### Study design and setting

MagPEP is a randomized, placebo-controlled phase III trial, in which adult patients with a clinical indication for diagnostic or therapeutic ERCP are randomized to receive either magnesium sulphate or placebo 60 min before and 6 hours after the intervention.

**Figure 1:**
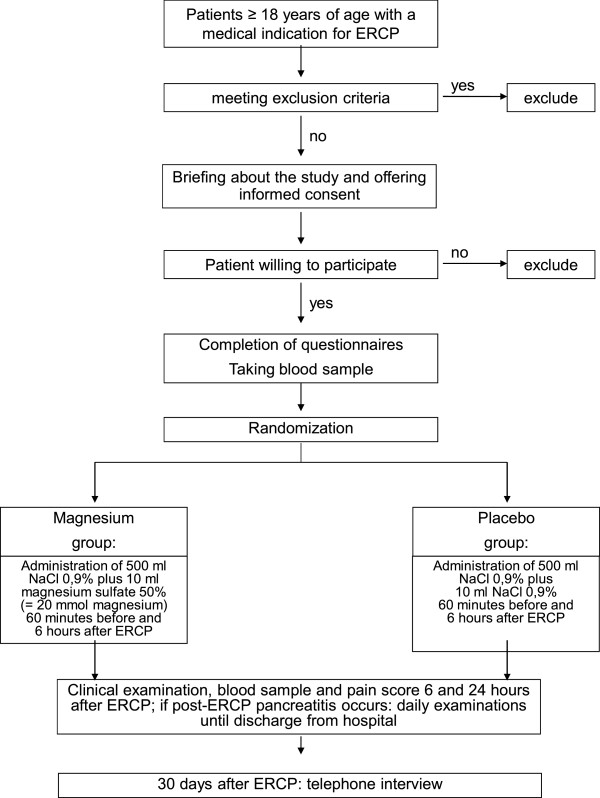
Randomization and treatment flow chart of the study.

The pathway by which patients are recruited and treated is shown in Figure 1.

### Patients

In all of the participating academic gastroenterology departments in Germany and Hungary, all adult (≥ 18 years of age) patients with a clinical indication for ERCP will be screened for eligibility for the trial. Exclusion criteria are listed in Table 1. Only patients who have not previously undergone an ERCP will be included in the study.

**Table 1:** Exclusion ciriteria

•	Previous ERCP
•	Known allergy or intolerance to one of the compounds used in the study
•	Participation in another interventional trial during the previous four weeks
•	Pregnancy and breast feeding
•	current acute pancreatitis
•	Renal insufficiency ≥ stage 4 (K/DOQI), i.e. MDRD-GFR < 30 ml/min/1,73 m^2^)
•	Hyperthyroidism
•	Symptomatic bradycardia <35 bpm
•	Atrioventricular block > 1° or other cardial conduction defects
•	Myasthenia gravis
•	Liver cirrhosis Child C
•	Overt coagulopathy
•	Kidney stone diathesis (calcium-magnesium-ammonium-phosphate stones)
•	Mental impairment, addiction or other disorders leading to the patients inability to understand the scope and possible consequences of a participation in a clinical trial
•	Magnesium medication within 14 days before the procedure
•	Inability to give informed consent

### Sample size considerations

Randomization of 502 patients (251 in each arm) will detect a 50% reduction in the relative risk of post ERCP pancreatitis in the treatment arm as compared to the placebo arm with 80% power (α-1 = 0.2) and a p-value of <0.05 (β-1 = 0.05). This figure assumes that the incidence of the primary end-point without preventive measures is 7.4%, which was the incidence of post-ERCP pancreatitis at the Department of Medicine A of Greifswald University Medicine in the period 2004–2005, and that the drop-out rate will be 5%.

#### Randomisation

Upon recruitment into the trial each patient will receive a unique identification number, making all patients identifiable for their local treating physician throughout the study. Only the local investigator will keep a personal list of patient numbers and patient names in order to recover records of the patients under his or her care after the end of the trial or for auditing purposes. After the patient's eligibility for randomisation has been assessed and written informed consent has been obtained, he/she will be randomly assigned to one of the two treatment arms (1:1 randomization) and receive a unique randomisation number, by which the order in which the patients are entered at the respective centre can be identified. Randomisation will be stratified by centre and reported to the PI-centre by fax within 24 h. The randomisation list will be kept under lock and key at the Department of Medicine A in Greifswald but will be accessible 24 hours a day in case of an emergency code break. Block randomization with blocks of four patients will be carried out centrally in the University Pharmacy Greifswald. The study is carried out in a double-blinded fashion.

### Treatment scheme

All randomized patients receive an intravenous infusion 60 min before the start and 6 hours after the end of ERCP. To prevent adverse reactions to magnesium, which are seen more frequently if the substance is injected rapidly, the first infusion is given over 60 min, the second infusion is given over 2 hours. In the magnesium arm 500 ml NaCl 0,9% containing 10 ml magnesium sulphate 50% (= 4930 mg magnesiumsulphate-heptahydrate; magnesium content: 486,1 mg = 20 mmol = 40 mval) will be administered. In the placebo arm 500 ml NaCl 0,9% + 10 ml NaCl 0,9% will be given in the same manner. Test substance vials are labelled with protocol and randomization number only giving no indication as to their content.

The ERCP intervention will be carried out according to the established clinical practice at the respective centre and in no way differently as compared to patients not participating in the study. After completion of the ERCP, the endoscopist is going to fill out a questionnaire on the details of the procedure, giving information e.g. on the final endoscopic diagnosis, the number of pancreatic duct injections, cannulation difficulties, the duration of the procedure, among other procedural details.

Six and 24 hours after ERCP patients are asked to assess their pain level using a visual analogue scale and the pain medication required and used is recorded. In addition, a clinical examination of the abdomen is performed and a blood sample is taken. To ensure blinding the clinician in charge of the patient is not allowed to order any tests on blood magnesium concentration unless in an emergency, which will lead to the exclusion of the patient. If post-ERCP pancreatitis develops blood samples, clinical examination, pain assessment and documentation of pain medication is continued until the day of discharge from the hospital.

### Follow-up

30 days after the ERCP all participants will be contacted by telephone in order to determine the 30-days morbidity and mortality as well as quality of life.

### Safety considerations

The assessment of safety will be based mainly on the frequency of adverse events (AEs) and on the number of laboratory values falling outside of pre-determined ranges. AEs will be summarized by presenting the number and percentage of patients having any AEs or serious adverse events (SAEs) and for each individual AE. Furthermore, the most common AEs (those occurring in at least 10% of the respective group) will be determined. Any other information collected (e.g. severity or relatedness to study drug) will be listed as appropriate. Laboratory data will be summarized by presenting shift tables using normal ranges (baseline to most extreme post-baseline value) and by presenting summary statistics of raw data and changes from baseline values (means, medians, standard deviations, ranges). The analysis of safety and tolerability will be based on all patients entered into treatment who received at least one application of the study medication. All proportions will be given along with exact Pearson-Clopper 95% confidence bounds.

### Statistical analysis

The primary analysis will be a comparison of magnesium versus placebo for the prevention of the primary end-point on an ‘intention to treat’ and ‘per protocol’analysis. Intention to treat and per protocol analyses will also be performed for the secondary outcomes. An interim analysis for monitoring safety issues and potential power calculation adjustments can be conducted by the data safety monitoring committee after half the patients have been recruited.

## Discussion

We report the protocol of a prospective randomized controlled trial to study the effectiveness of magnesium sulphate in the prevention of post-ERCP pancreatitis, the most important complication of diagnostic and therapeutic ERCP. Previous *in vitro* experiments and animal studies on the role of magnesium in the pathophysiology of acute pancreatitis have suggested that magnesium may be an effective compound to prevent post-ERCP pancreatitis. After various but mostly ineffective substances have been tested in the prophylaxis of post-ERCP pancreatitis, magnesium sulphate could have the potential for a wide introduction into clinical practise: 1) Its administration is simple and allows for outpatient treatment. 2) Adverse effects are rare and usually mild [24]. 3) Contraindications are minimal. 4) Costs are minimal and would be more then recovered by a reduction in post-ERCP pancreatitis.

The intravenous magnesium sulphate dosage used in the MagPEP-study was chosen on the basis of previous trials investigating the effect of magnesium sulphate on multiple other conditions, including asthma, traumatic brain injury, acute myocardial infarction, premature labour, and stroke. Data from several hundred patients have demonstrated that magnesium sulphate in the proposed dosing is not likely to cause severe adverse effects, which makes the study ethically justifiable, feasible and safe for the participants.

## Conclusions

If magnesium sulphate is found to be effective in the prevention of post-ERCP pancreatitis, this inexpensive compound with minimal side effects could become a routine pharmacological prophylaxis for post-ERCP pancreatitis.

## Abbreviations

AE: Adverse Events; CRF: Case Report Form; CRO: Contract Research Organisation; DSMB: Data and Safety Monitoring Board; ERCP: Endoscopic retrograde cholangiopancreatography; GCP: Good Clinical Practice; i.v: Intravenous; NaCl: Sodium chloride; QoL: Quality of Life; SAE: Serious Adverse Events

## Competing interests

The authors declare that they have no competing interests.

## Authors’ contributions

JM, GF and MML have designed the study, TK performed power calculations and pre-study statistical analysis, GE is in charge of randomization and blinding of placebo and verum trial medication, EW runs the central trial centre and database, all other authors serve as trial investigators, JM and GF obtained central ethics approval and MML has overall legal responsibility on behalf of sponsors. All authors read and approved the final manuscript.

## Pre-publication history

The pre-publication history for this paper can be accessed here:

http://www.biomedcentral.com/1471-230X/13/11/prepub
